# The Angry Patient: CBT-Informed De-escalation and Emotional Regulation Protocol in Mental Health Practice

**DOI:** 10.1192/j.eurpsy.2026.11738

**Published:** 2026-07-31

**Authors:** A. Argyriadis, A. Argyriadi

**Affiliations:** 1 Hellenic Mediterranean University, Evidence based Health, Education and Clinical Protocols Laboratory, Heraklion, Greece; 2 Frederick University, Nicosia, Cyprus

## Abstract

**Introduction:**

Aggressive behaviors among psychiatric inpatients remain a major clinical and safety concern for nursing staff, often exacerbating patient distress and impeding therapeutic engagement. While pharmacological management is often emphasized, non-pharmacological strategies such as Cognitive Behavioral Therapy (CBT)-informed protocols for de-escalation and emotional regulation offer an evidence-based, person-centered alternative.

**Objectives:**

The objectives of this case study are to present a CBT-informed de-escalation protocol applied in the management of acute anger in a psychiatric patient, highlighting the structured use of cognitive and behavioral techniques in crisis situations. It further aims to explore the pivotal role of mental health nurses in implementing emotion regulation strategies grounded in CBT, emphasizing their unique position to translate psychological principles into day-to-day clinical practice. Finally, the study seeks to evaluate the outcomes of a non-pharmacological, evidence-based intervention in reducing aggression and enhancing patient engagement, thereby underscoring the potential of nursing-led CBT approaches to promote safer therapeutic environments and foster meaningful patient participation in care.

**Methods:**

A single-case study design was employed, involving a 37-year-old male inpatient with schizoaffective disorder and a history of impulsive aggression. Nursing staff were trained to apply a three-step CBT-informed protocol. More specifically, identification of triggers and automatic thoughts, implementation of grounding and de-escalation techniques (deep breathing, cognitive reframing, and use of cue cards), structured post-incident reflection using ABC (Antecedent-Behavior-Consequence) charts and Pre- and post-intervention assessments included the Modified Overt Aggression Scale (MOAS) and nurse-reported therapeutic alliance scores.

**Results:**

Within two weeks, a marked reduction in aggressive outbursts was observed, with MOAS scores dropping from 10 to 3. Health professionals reported improved patient cooperation and a stronger therapeutic bond. The patient demonstrated growing insight into his emotional triggers and actively engaged in self-monitoring.

**Conclusions:**

CBT-informed emotional regulation protocols can be successfully implemented by psychiatric nurses as part of routine care, offering a structured, non-pharmacological response to patient aggression. The integration of CBT principles into de-escalation strategies enhances both patient safety and empowerment, while supporting nursing staff in managing complex emotional behaviors.

**Disclosure of Interest:**

None Declared

